# CT-free kidney single-photon emission computed tomography for glomerular filtration rate

**DOI:** 10.1038/s41598-025-12595-2

**Published:** 2025-07-25

**Authors:** Kyounghyoun Kwon, Dongkyu Oh, Ji Hye Kim, Jihyung Yoo, Won Woo Lee

**Affiliations:** 1 Department of Health Science and Technology, Graduate School of Convergence Science and Technology, Seoul National University, 145 Gwanggyo-ro, Yeongtong- gu, Suwon-si, Gyeonggi-do 16229 Republic of Korea; 2 Department of Nuclear Medicine, Seoul National University Bundang Hospital, 82 Gumi-ro, 173 Beon-gil, Bundang-gu, Seongnam-si, Gyeonggi-do 13620 Republic of Korea; 3 BioDrone Research Institute, MDimune Inc, 49 Achasan-ro, Seongdong-gu, Seoul, 04790 Republic of Korea; 4 Department of Nuclear Medicine, Seoul National University College of Medicine, 103 Daehak-ro, Jongno-gu, Seoul, 03080 Republic of Korea; 5 Institute of Radiation Medicine, Medical Research Center, Seoul National University, 101 Daehak-ro, Jongno-gu, Seoul, 03080 Republic of Korea

**Keywords:** Deep-learning, Organ segmentation, Kidney, SPECT/CT, Glomerular filtration rate, Medical research, Nephrology

## Abstract

This study explores an artificial intelligence-based approach to perform CT-free quantitative SPECT for kidney imaging using Tc-99 m DTPA, aiming to estimate glomerular filtration rate (GFR) without relying on CT. A total of 1000 SPECT/CT scans were used to train and test a deep-learning model that segments kidneys automatically based on synthetic attenuation maps (µ-maps) derived from SPECT alone. The model employed a residual U-Net with edge attention and was optimized using windowing-maximum normalization and a generalized Dice similarity loss function. Performance evaluation showed strong agreement with manual CT-based segmentation, achieving a Dice score of 0.818 ± 0.056 and minimal volume differences of 17.9 ± 43.6 mL (mean ± standard deviation). An additional set of 50 scans confirmed that GFR calculated from the AI-based CT-free SPECT (109.3 ± 17.3 mL/min) was nearly identical to the conventional SPECT/CT method (109.2 ± 18.4 mL/min, *p* = 0.9396). This CT-free method reduced radiation exposure by up to 78.8% and shortened segmentation time from 40 min to under 1 min. The findings suggest that AI can effectively replace CT in kidney SPECT imaging, maintaining quantitative accuracy while improving safety and efficiency.

**Supplementary Information:**

The online version contains supplementary material available at 10.1038/s41598-025-12595-2.

## Introduction

Glomerular filtration rate (GFR) is a critical biomarker of kidney function and is closely associated with the morbidity and mortality of patients with chronic kidney disease (CKD)^1,2^. Accurate measurement of GFR is important for managing patients at risk of CKD, especially those with underlying conditions such as diabetes. The administration of therapeutic agents in various clinical settings is strongly influenced by the GFR level^3,4^. In addition to total GFR, the individual kidney GFR—particularly after nephrectomy - serves as a key prognostic indicator in patients with kidney tumors and in kidney donors^5,6^. Although planar gamma camera scans have traditionally been used to measure individual kidney GFR^7,8^their accuracy has been questioned due to limited capabilities in correcting for radioactivity^9,10^.

Quantitative single-photon emission computed tomography/computed tomography (SPECT/CT) is an evolving technology in nuclear medicine, where CT plays crucial roles in attenuation correction (AC) and organ segmentation (OS)^11^. CT provides an attenuation map (µ-map) that is critical for the AC of photons, and serves as a canvas for OS^12,13^. The organ of interest is segmented and then applied to the quantitative SPECT to produce clinically useful parameters, such as %injected dose (%ID) or standardized uptake value (SUV)^14–16^. The utility of quantitative SPECT/CT ranges from diagnosis of Tc-99 m based nuclear imaging tests to treatment planning of radioactive iodine for thyroid diseases^17–27^.

Kidney SPECT/CT using Tc-99 m diethylenetriaminepentaacetic acid (DTPA) is one of the applications of quantitative SPECT/CT^10,28^. The %ID by the SPECT/CT measured at the time of 2–3 min post administration of Tc-99 m DTPA could be converted to glomerular filtration rate (GFR), which has better reproducibility and accuracy compared to the conventional planar imaging-based GFR^9,10^.

One application of artificial intelligence (AI) in the field of nuclear medicine is to deprive SPECT/CT of CT^29,30^. This would minimize radiation exposure to patients and reduce human efforts for OS. In thyroid SPECT/CT, CT roles (i.e., the µ-map generation and thyroid segmentation) were completely replaced by deep learning (DL), resulting in CT-free thyroid SPECT^31^.

In this study, we attempted to achieve CT-free kidney SPECT for GFR, substituting conventional kidney SPECT/CT. We focused on automatic kidney segmentation using synthetic µ-maps generated from SPECT images^32^. We aimed to ameliorate CT-induced radiation exposure to patients, and human resources for kidney segmentation.

## Materials and methods

The study has been approved by the institutional review board of the Seoul National University Bundang Hospital (Seoul National University Bundang Hospital Institutional Review Board No: B-2505-970-102). The need for written informed consent was waived by the institutional review board of the Seoul National University Bundang Hospital. All methods in the current research were performed in accordance with the national guidelines following the Declaration of Helsinki^33^ and regulations following the ICMJE (International committee of medical journal editors)^34^.

### Dataset

Two different datasets of Tc-99 m DTPA SPECT/CT from December 2019 to April 2021 were utilized in the study (Table 1). The first dataset (*n* = 1000) catered to the development of a DL algorithm for the automatic kidney segmentation, whereas the second dataset (*n* = 50) served to verify the developed algorithm. There was no difference of characteristics between the two datasets (Table 1). Details of the acquisition and reconstruction parameters of the kidney SPECT/CT are provided in the supplementary material.

**Table 1:** Characteristics of kidney SPECT/CT datasets.

		For development of CT-free SPECT (*n* = 1000)	For verification of the CT-free SPECT (*n* = 50)	*P* value
Sex (male: female)		690:310	37:13	0.4549
Age (years)		56.8 ± 13.2	58.9 ± 13.8	0.2687
Height (cm)		166.1 ± 9.5	167.3 ± 7.6	0.3306
Weight (kg)		70.7 ± 13.7	71.6 ± 11.8	0.5955
BSA* (m^2^)		1.78 ± 0.20	1.80 ± 0.17	0.4017
Reason for SPECT/CT	Normal (kidney donor)	10	1	0.6190
	Renal tumor	307 (Rt: Lt: Both = 151:151:5)	11 (Rt: Lt: Both = 7:4:0)	
	Urinary stone	151 (Rt: Lt: Both = 47:55:49)	7 (Rt: Lt: Both = 4:2:1)	
	Post partial nephrectomy	516 (Rt: Lt: Both = 261:244:11)	30 (Rt: Lt: Both = 12:17:1)	
	Post total nephrectomy	8 (Rt: Lt = 6:2)	1 (Rt: Lt = 0:1)	
	Others	8^†^	0	

*BSA; body surface area by the Dubois formula:

BSA (m2) = 0.007184 x weight (kg)0.425 x height (cm)0.725.

^†^Others include 3 ureter tumors (Rt: Lt = 1:2), 3 ureter stricture (Rt: Lt = 3:0), and 2 horse-shoe kidneys with renal stone (Rt: Lt = 0:2).

Data are mean ± standard deviation.

The 1000 SPECT/CT cases of the first dataset were allocated in an 8:1:1 ratio for training, validation, and testing of the kidney segmentation algorithm (Supplemental Table 1).

### Study scheme

**Fig. 1:**
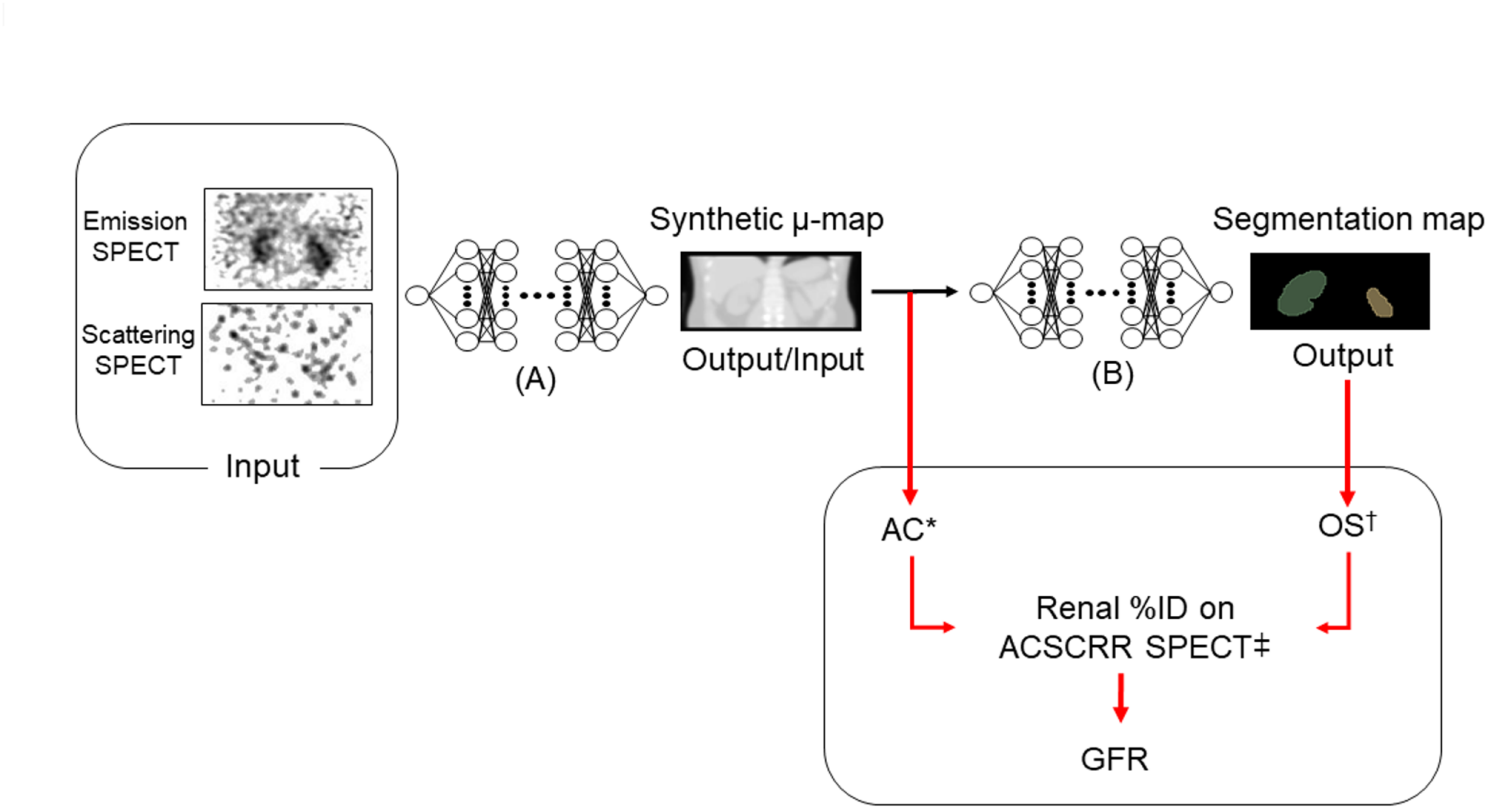
Study scheme of CT-free GFR SPECT. (**A**) Synthetic µ-map generator and (**B**) automatic kidney segmentation algorithm (AC*, attenuation correction; OS^†^, organ segmentation; ACSCRR SPECT^‡^, quantitative SPECT with attenuation correction, scatter correction, and resolution recovery applied; and GFR, glomerular filtration rate).

The CT-free SPECT for GFR comprised of two separate convolutional neural networks (CNNs) (Fig. 1). The first algorithm, which had been previously reported by our group^32^was a network that took SPECT images (including both primary emission and scatter) as inputs to generate synthetic µ-map outputs, which were then used as inputs for the second segmentation algorithm. The resultant µ-map facilitated the AC of the emission SPECT, leading to a quantitative SPECT in conjunction with scatter correction (SC) and resolution recovery (RR). The DL algorithm for the automatic kidney segmentation used the synthetic µ-map as input, and the manual kidney segmentation map as its label. Two experts (RN. JHK and RN. JY), who were nuclear medicine specialists for OS, provided the kidney segmentation map as label. They drew multiple regions-of-interest (ROIs) on coronal CT images of the respective SPECT/CT until the whole volume of kidneys was included. At least 40 min per case were required to obtain the volumes-of-interest (VOIs) for the bilateral kidneys (Supplemental Fig. 1).

The output of the kidney segmentation algorithm was applied to the quantitative ACSCRR SPECT, yielding %ID of Tc-99 m DTPA over the segmented kidneys. The %ID of Tc-99 m DTPA was then converted to GFR using the equation we had previously reported: GFR (mL/min) = 9.1462 x %ID + 23.0653^10^.

### Preprocessing for deep-learning of µ-map generation

To generate µ-maps, SPECT images (both primary emission and scattering) were used as inputs for the synthetic µ-map generator (Fig. 1). Logarithmic maximum normalization was applied to the input SPECTs. Here, primary emission SPECT refers to the SPECT image reconstructed using primary photons at a 140 keV energy peak (20% window: 126–154 keV), whereas scattering SPECT refers to the SPECT image reconstructed using scattered photons at a 120 keV energy peak (10% window: 115–125 keV). The primary emission and scattering SPECT images were reconstructed from the respective sinograms using vendor-provided software (Q. VolumetrixMI, GE Healthcare) with correction for the collimator-detector response (i.e., resolution recovery, RR), resulting in NCRR SPECT. Here, NC indicates neither attenuation correction nor scatter correction. Ordered subset expectation maximization (OSEM) algorithm was used for the SPECT reconstruction with 10 subsets and 4 iterations. For statistical noise reduction, a Butterworth low-pass filter (order of 10 and cutoff frequency of 0.48 cycles/cm) was applied to the scattering SPECT images^32^. SPECT image matrix was 128 × 128 × 128 and voxel size was 3.45 × 3.45 × 3.45 mm^3^. CT images were reconstructed into 512 × 512 × 161 matrix and 0.977 × 0.977 × 2.5 mm^3^ voxel size.

### Preprocessing for deep-learning of kidney segmentation

The generated synthetic µ-maps were normalized in two ways. One method involved maximum normalization. The other used a windowing-maximum normalization technique^35^which first constrained the attenuation coefficients within a range of [0, w] with w being 0.3, 0.4, and 0.5, followed by the maximum normalization. Initially, the matrix and voxel sizes were 64 × 128 × 128 and 3.45 × 3.45 × 3.45 mm^3^respectively, for the synthetic µ-maps and manual segmentation maps. To reduce training time, the matrix was cropped to 64 × 64 × 96. The manual segmentation map was smoothed through morphological closing operation.

### Network architecture for µ-map generation

A 3D U-net with 64 initial neurons and 4 skip connections was used. The architecture employed instance normalization and nearest-neighbor interpolation because of their proven capability for µ-map generation^32^. The details are seen in the Supplemental Fig. 2.

### Network architecture for kidney segmentation

**Fig. 2:**
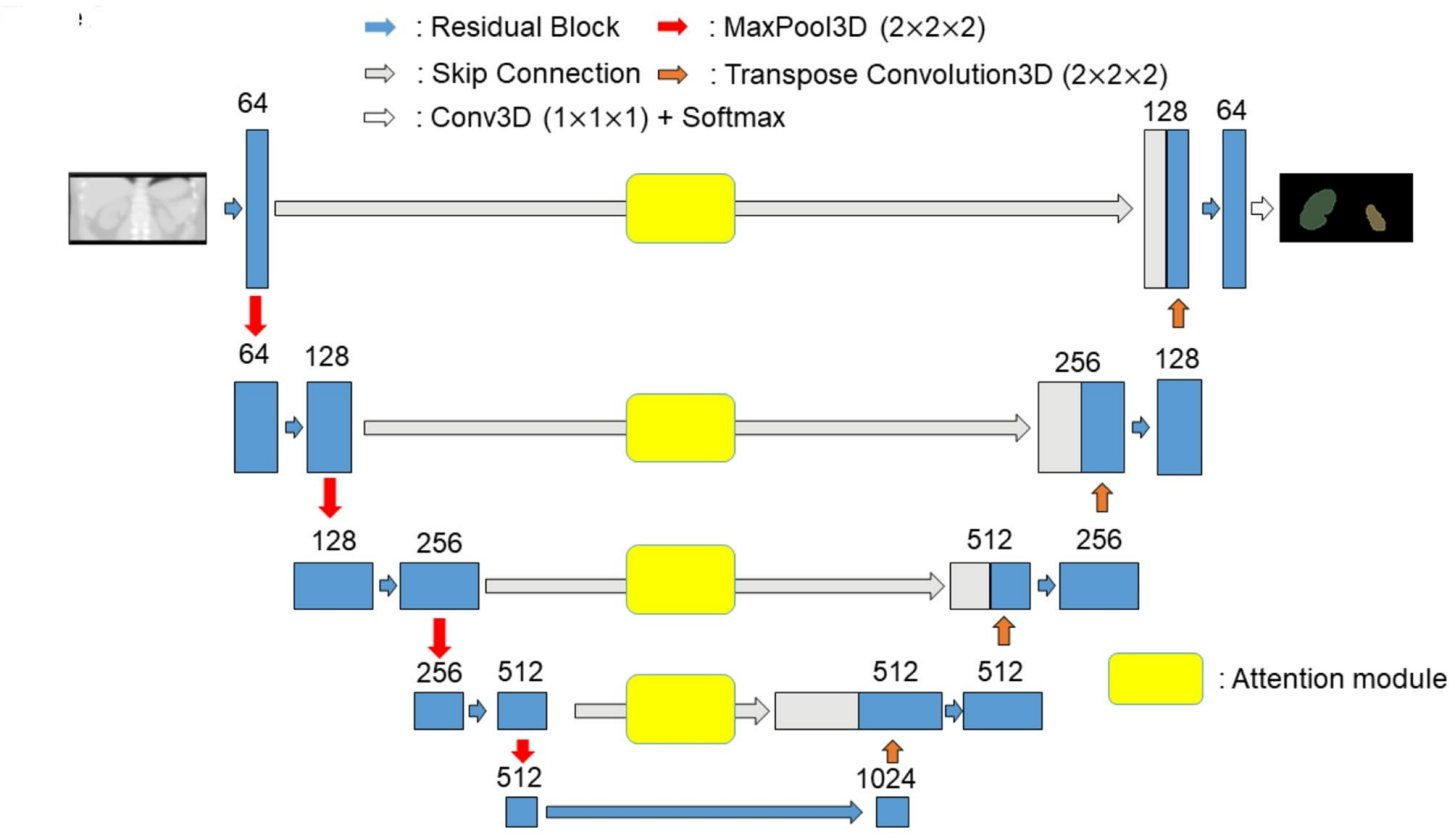
Model architecture for the automatic kidney segmentation.

The 3-dimensional U-net with 64 initial neurons and 4 skip connections was employed for the kidney segmentation algorithm (Fig. 2). Common CNN architecture with residual block (residual U-Net) was investigated^36^. Edge attention module was introduced during skip connection in order to detect/emphasize the edge of the kidneys^37^ (Supplemental Fig. 3).

We trained our networks using TensorFlow^38^ and the Keras framework^39^.

### Loss function

The loss function for the automatic kidney segmentation was a categorical cross-entropy (CCE) loss or a generalized Dice similarity coefficient (GDSC) loss, which were defined as follows:

$$\:\text{C}\text{C}\text{E}=-\frac{1}{n}\sum\:_{i=1}^{n}[{y}_{i}log\left({\widehat{y}}_{i}\right)+\left(1-{y}_{i}\right)log(1-{\widehat{y}}_{i})]$$

$$\:\text{G}\text{D}\text{S}\text{C}=1-\frac{2{\sum\:}_{i}^{n}{w}_{i}\left[{y}_{i}{\widehat{y}}_{i}\right]}{{\sum\:}_{i}^{n}{w}_{i}\left[{y}_{i}\right]+{\sum\:}_{i}^{n}{w}_{i}\left[{\widehat{y}}_{i}\right]}$$

where $\:{y}_{i}$ was 0 or 1 as the ground truth, $\:{\widehat{y}}_{i}$ was the probability of prediction, *w*_*i*_ was the weighting factor for class *i*, and n was the number of classes. The GDSC loss was used instead of the standard DSC loss to reduce the impact of class imbalance (i.e., greater size of background compared to smaller kidneys), and represented by one minus the weighted average DSC score^40^. The number of classes was either 3 (i.e., right kidney, left kidney, and background) or 2 (i.e., kidneys and background).

### Training hyper-parameters

Training epochs were set to 100 with a batch size of eight. Early stopping rules were applied during the initial 10 epochs. The adaptive moment estimation optimizer was used with a learning rate of 0.001 and an exponential decay rate of 0.96. Flip augmentation was applied along the x and z axes. The training time was approximately 15 min per epoch. The computer hardware for network training was AMD Ryzen7 5800X CPU (AMD Inc., Santa Clara, CA, USA) and Nvidia RTX 3090 GPU (Nvidia Corp., Santa Clara, CA, USA).

### Metrics for outcome evaluations

To investigate the outcomes of the automatic kidney segmentation algorithm, Dice similarity coefficient (DSC) (see supplementary material for details) and volume difference (VD) (= ground truth – predicted volumes) were employed.

For the performance evaluation of the synthetic µ-map generator, R^2^mean squared error (MSE), and %normalized mean absolute error (%NMAE) were used to evaluate the performance of the synthetic µ-map generator (see supplementary material for details).

### Training sequence

The baseline training conditions for the automatic kidney segmentation were (1) the synthetic µ-map input from the µ-map generator, (2) maximum normalization of the µ-map input, (3) conventional U-Net as training framework, (4) CCE difference as loss function and (5) 3 classes for segmentation (i.e., right kidney, left kidney, and background).

We initially investigated whether SPECT support, additional to the µ-map input, could improve the performance of kidney segmentation. Next, we explored the application of windowing-maximum normalization of the synthetic µ-map input^35^. Next, the 3-dimensional GDSC loss function was tested^40^. Finally, modifications of the U-Net architecture (from convolutional block to residual block) and addition of edge attention module during skip connection were tested about the performance improvement.

### Statistical analysis

Parametric analyses (i.e., student *t* and analysis-of-variance tests) were performed for continuous variables when the Shapiro–Wilk test did not reject normal distribution features. Otherwise, nonparametric tests (i.e., Mann–Whitney and Kruskal–Wallis tests) were performed. Categorical variables were compared using the chi-square test. GFR was compared using the paired *t*-test between CT-free SPECT and conventional SPECT/CT. Statistical significance was set at *p* < 0.05. All analyses were performed using MedCalc (version 23.1.1; Ostend, Belgium).

## Results

### SPECT input support

The tested SPECTs were the primary emission SPECT (P) alone, the combination of primary emission SPECT and scattering SPECT (PS), and the quantitative ACSCRR SPECT (Q). Here, ACSCRR means correction for attenuation, scattering, and collimator-detector response variation (i.e., resolution recovery). We normalized P, PS, and Q by either maximum or logarithmic maximum normalization (see supplementary material for details). DSC and VD were employed as the metric for the DL outcomes. As a result, the SPECT input support, in addition to the synthetic µ-map input, did not improve the task of kidney segmentation. The range of DSC was broad with complete segmentation failure often observed, and VD was unacceptably high, indicating significant underestimation of kidney volume (Supplemental Table 2). Hereafter, µ-map input alone was applied to the following investigations.

### Normalization of the µ-map input, loss function, and number of classes

Next, we investigated the normalization of the µ-maps (windowing-maximum vs. maximum), loss functions (CCE vs. GDSC), and number of classes (3 vs. 2). The upper limit of the µ-map attenuation coefficients during the windowing-maximum normalization was constrained to 0.5 (Supplemental Table 3).

**Fig. 3:**
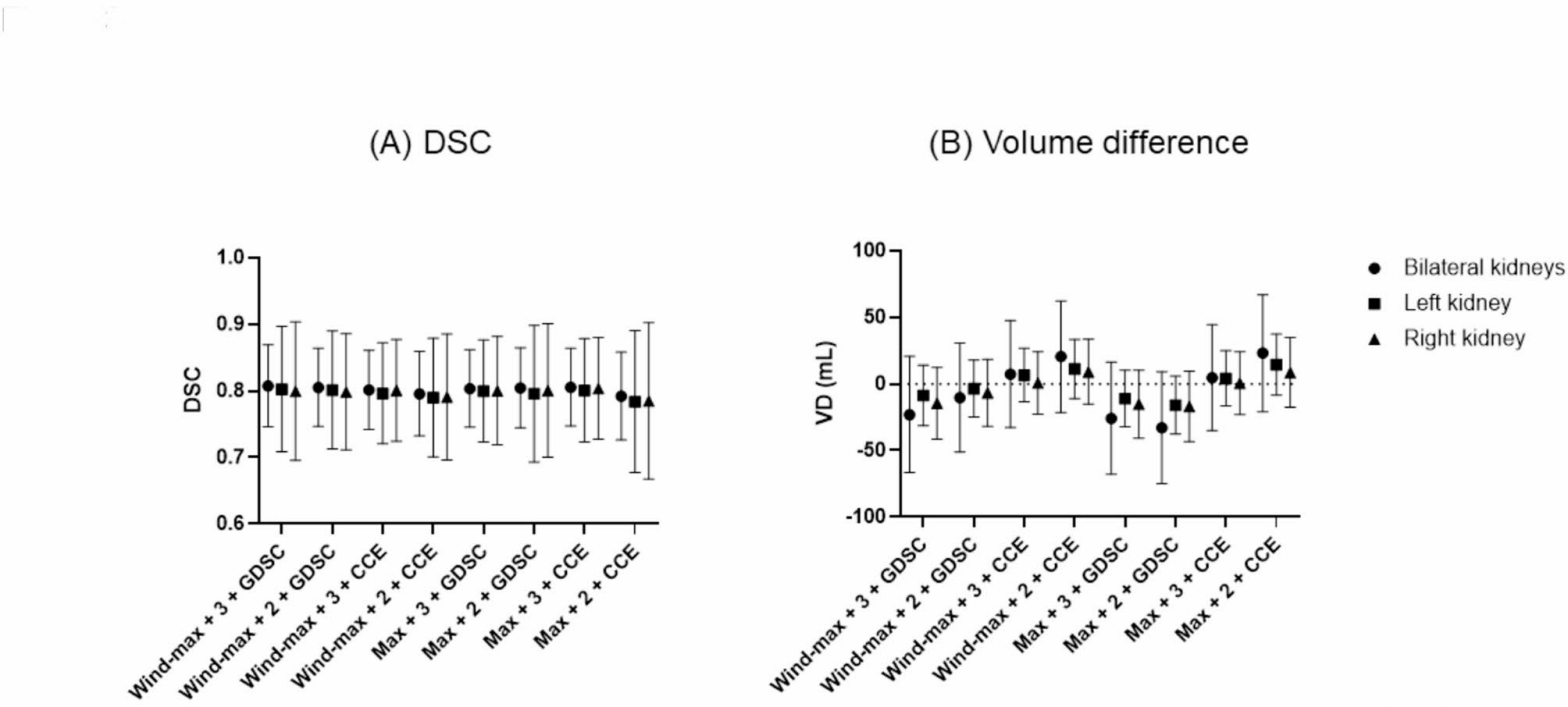
The performances of automatic kidney segmentation according to the input normalization (windowing-maximum normalization vs. maximum normalization), loss function (CCE loss vs. GDSC loss), and number of segmentation classes (3 vs. 2). (**A**) DSC, and (**B**) volume difference.

The highest DSC was observed in a combination of windowing-maximum normalization, GDSC loss function, and use of 3 segmentation classes (Table 2; Fig. 3). VD using the combination was slightly negative, indicating that the predicted volume of kidneys was slightly greater than the ground truth. The main task of kidney segmentation should be minimizing the loss of radioactivity. Thus, the greater predicted kidney volumes (i.e., negative VD) was favored over the smaller predicted kidney volumes (i.e., positive VD).

**Table 2:** The performance by normalization of the µ-map input, loss function, and number of classes (*n* = 100 testing cases). (input = µ-map only, CNN model = U-Net)

µ-map normalization + no. of classes	Loss function	DSC (total)	DSC (left)	DSC (right)	VD in mL (total)	VD in mL (left)	VD in mL (right)
Wind-max + 3	GDSC	0.808 ± 0.061 (0.501–0.893)	0.803 ± 0.094 (0.144–0.910)	0.800 ± 0.104 (0.152–0.902)	− 23.0 ± 43.6 (− 6.0–3.7)	− 8.6 ± 22.8 (− 2.9–3.0)	− 14.4 ± 27.0 (− 3.2–3.6)
Wind-max + 2	GDSC	0.805 ± 0.059 (0.502–0.884)	0.801 ± 0.089 (0.206–0.901)	0.799 ± 0.087 (0.350–0.884)	− 10.2 ± 40.9 (− 4.2–3.8)	− 3.6 ± 21.4 (− 2.3–2.9)	− 6.6 ± 25.0 (− 2.7–3.2)
Wind-max + 3	CCE	0.802 ± 0.059 (0.512–0.886)	0.796 ± 0.076 (0.375–0.897)	0.801 ± 0.076 (0.444–0.900)	7.4 ± 40.1 (− 4.6–4.5)	6.7 ± 20.1 (− 2.1–2.9)	0.7 ± 23.4 (− 2.5–2.7)
Wind-max + 2	CCE	0.796 ± 0.063 (0.490–0.881)	0.790 ± 0.089 (0.216–0.896)	0.791 ± 0.095 (0.332–0.889)	20.4 ± 41.8 (− 3.5–4.9)	11.2 ± 22.1 (− 1.7–2.9)	9.1 ± 24.2 (− 1.9–3.9)
Max + 3	GDSC	0.804 ± 0.058 (0.510–0.879)	0.800 ± 0.076 (0.407–0.902)	0.800 ± 0.081 (0.365–0.901)	− 26.0 ± 42.0 (− 5.9–3.2)	− 10.9 ± 21.2 (− 3.7–1.3)	− 15.1 ± 25.6 (− 2.5–3.3)
Max + 2	GDSC	0.804 ± 0.060 (0.487–0.881)	0.796 ± 0.103 (0.000–0.904)	0.801 ± 0.100 (0.120–0.893)	− 32.8 ± 42.1 (− 5.4–4.4)	− 15.9 ± 21.8 (− 2.9–3.6)	− 17.0 ± 26.5 (− 2.8–4.9)
Max + 3	CCE	0.805 ± 0.058 (0.495–0.885)	0.801 ± 0.077 (0.343–0.894)	0.804 ± 0.076 (0.432–0.897)	4.7 ± 39.7 (− 4.2–4.3)	4.2 ± 20.6 (− 1.8–2.6)	0.5 ± 23.4 (− 2.4–2.6)
Max + 2	CCE	0.792 ± 0.066 (0.484–0.879)	0.784 ± 0.107 (0.000–0.890)	0.785 ± 0.117 (0.000–0.885)	23.1 ± 43.9 (− 3.3–6.4)	14.4 ± 22.8 (− 1.3–3.6)	8.6 ± 26.1 (− 2.0–5.3)

Wind-max, windowing then maximum normalization; Max, maximum normalization; GDSC, generalized Dice similarity coefficient; CCE, categorical cross-entropy; VD, volume difference; DSC, Dice similarity coefficient.

Date are range (mean ± standard deviation).

The use of maximum normalization instead of windowing-maximum normalization caused slightly lower DSC. The employment of CCE rather than GDSC elicited smaller predicted volumes. The two classes tended to generate lower DSC and smaller predicted volumes, compared to the three classes (Table 2; Fig. 3).

### Selection of model architecture

**Fig. 4:**
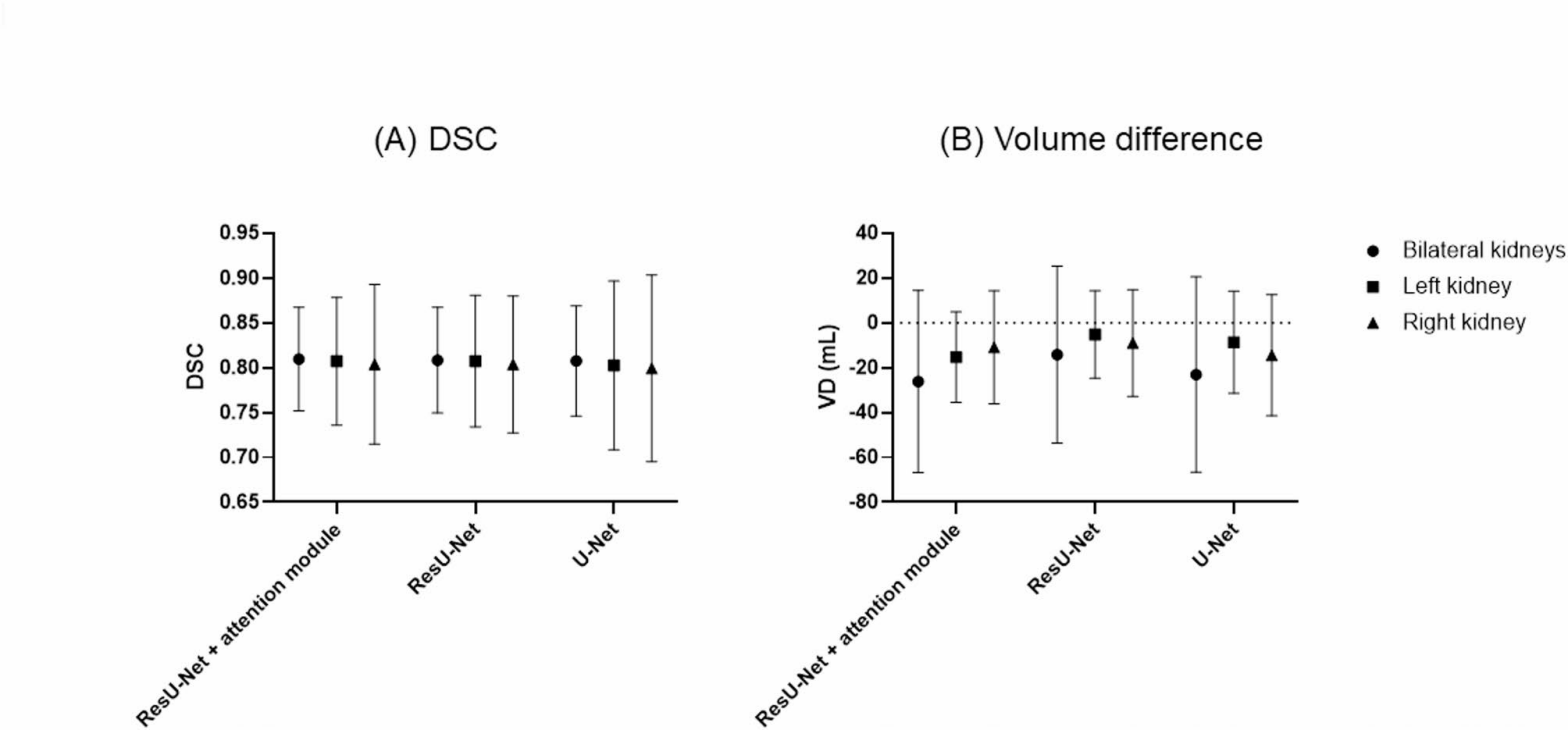
The performances of automatic kidney segmentation according to the model architecture (U-Net vs. ResU-Net vs. ResU-Net with attention module). (**A**) DSC, and (**B**) volume difference.

Finally, we investigated the CNN architecture among the conventional U-Net, residual U-Net, and residual U-Net plus attention module applications (Fig. 2 and Supplemental Fig. 3). VD was negative in all occasions without significant difference but DSC was the highest with the model architecture of ResU-Net with attention module application (Table 3; Fig. 4).

**Table 3:** The performance by CNN model architecture (*n* = 100 testing cases). (input = µ-map only, normalization = windowing then maximum normalization, loss function = gdsc loss, number of classes = 3).

CNN model	DSC	Left DSC	Right DSC	VD in mL (total)	VD in mL (left)	VD in mL (right)
ResU-Net + attention module	0.810 ± 0.058 (0.514–0.894)	0.807 ± 0.071 (0.437–0.910)	0.804 ± 0.089 (0.317–0.895)	− 26.1 ± 40.7	− 15.2 ± 20.2	− 10.9 ± 25.2
ResU-Net	0.809 ± 0.059 (0.509–0.898)	0.808 ± 0.073 (0.437–0.911)	0.804 ± 0.076 (0.457–0.890)	− 14.1 ± 39.5	− 5.1 ± 19.5	− 9.0 ± 23.9
U-Net	0.808 ± 0.061 (0.501–0.893)	0.803 ± 0.094 (0.144–0.910)	0.800 ± 0.104 (0.152–0.902)	− 23.0 ± 43.6	− 8.6 ± 22.8	− 14.4 ± 27.0

GDSC, generalized Dice similarity coefficient; ResU-Net, residual U-Net; VD, volume difference; DSC, Dice similarity coefficient.

Date are range (mean ± standard deviation).

### Deep-learning algorithm for µ-map generation

**Fig. 5:**
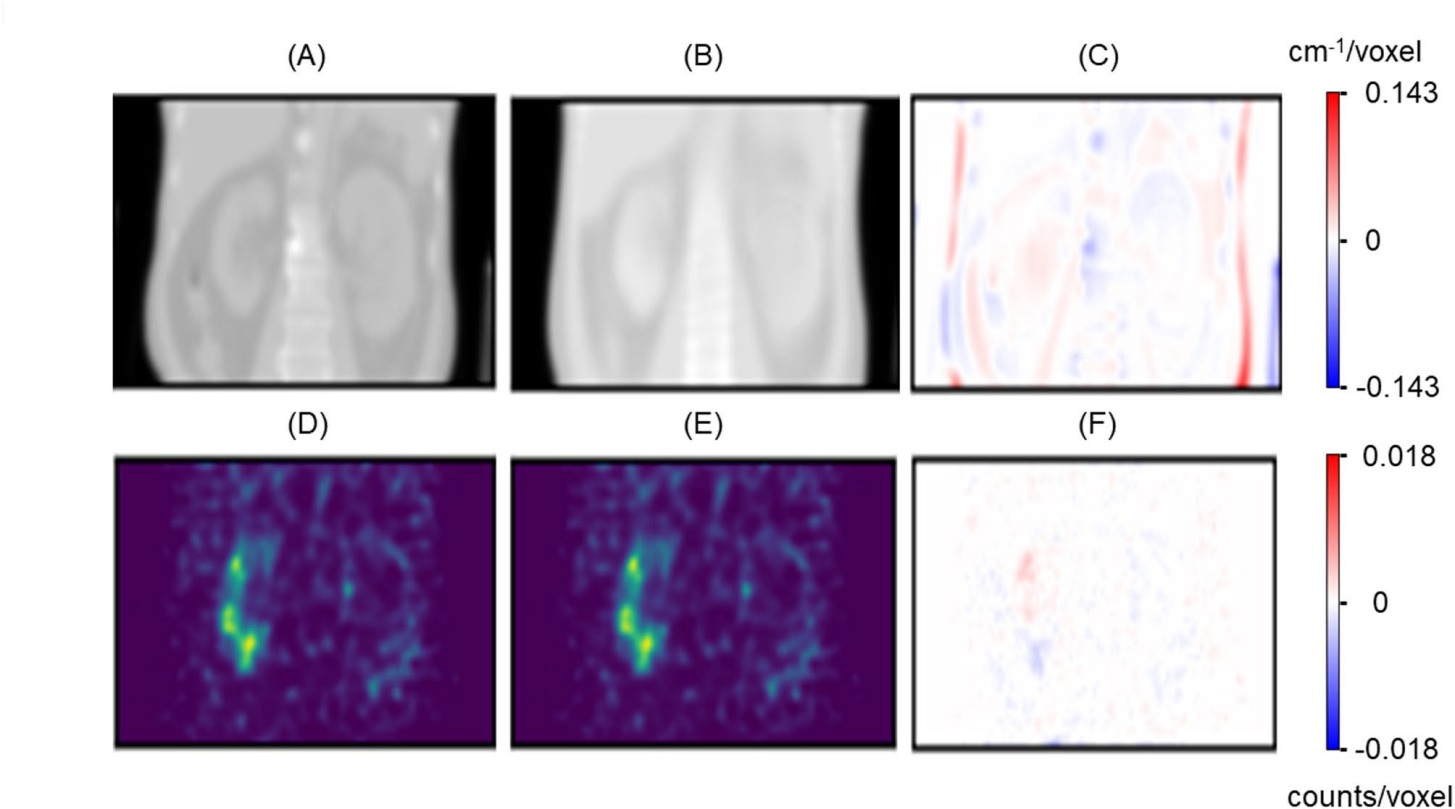
Comparison of CT-derived and synthetic µ-maps and their impact on reconstructed SPECT images. Upper row: (**A**) CT-derived µ-map, (**B**) synthetic µ-map generated by the µ-map generator network, and (**C**) voxel-wise difference between (**A**) and (**B**). Lower row: (**D**) SPECT image reconstructed using CT-derived µ-map, (**E**) SPECT image by synthetic µ-map, and (**F**) voxel-wise difference between (**D**) and (**E**). The µ-map differences lead to minor variation in the reconstructed SPECT images, primarily around anatomical edges, indicating the synthetic µ-map’s suitability for attenuation correction in SPECT. Color bars indicate attenuation coefficients (cm^− 1^/voxel) and reconstructed counts (counts/voxel), respectively.

The synthetic µ-maps produced by the first µ-map generator were visually indistinguishable from the CT-derived µ-maps. The SPECT images reconstructed using the synthetic µ-maps showed only minor differences compared to those reconstructed using CT-derived µ-maps (Fig. 5).

### Deep-learning algorithm for automatic kidney segmentation

**Fig. 6:**
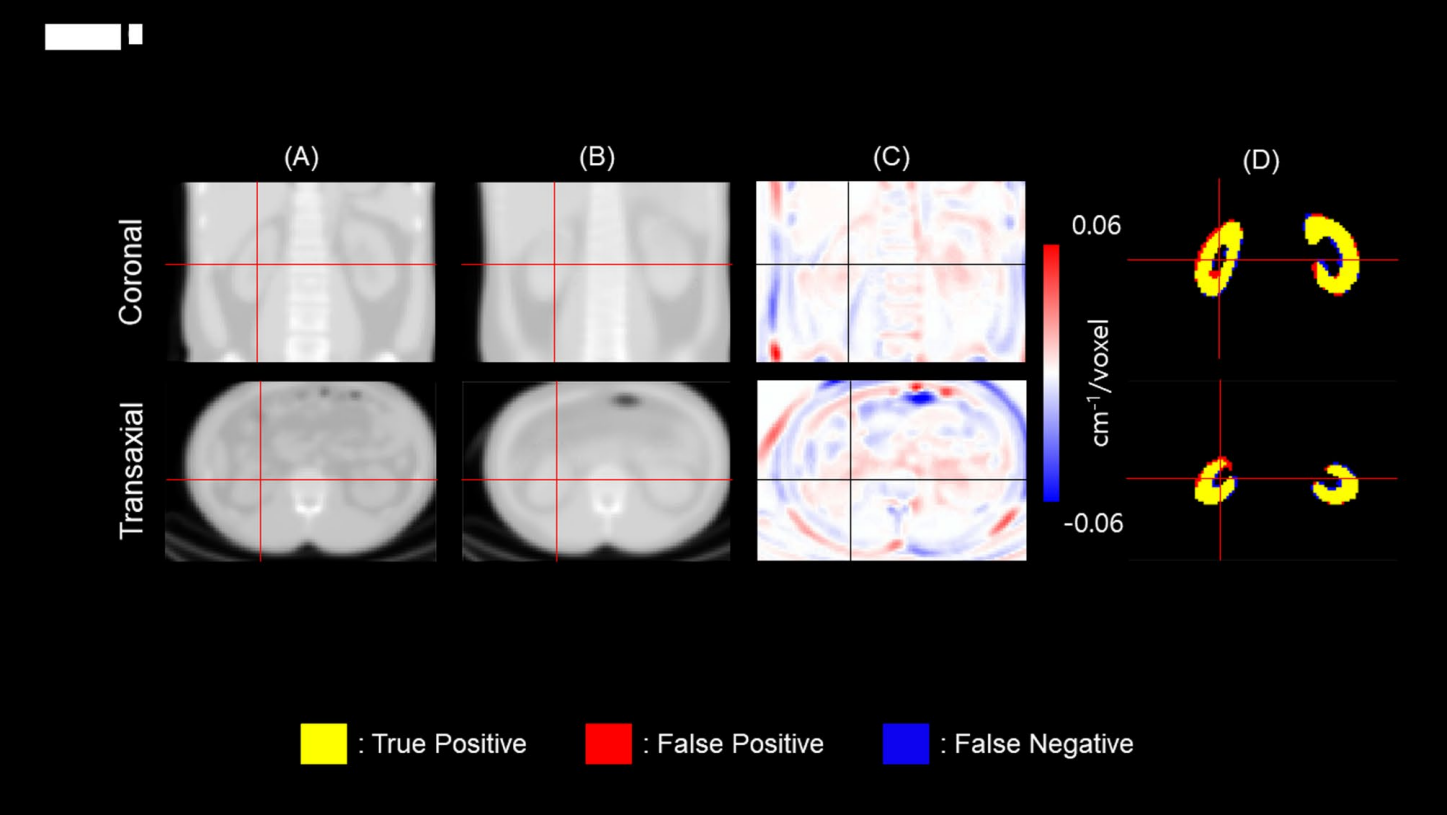
Demonstration of automatic kidney segmentation. (**A**) CT-derived µ-map as the reference, (**B**) synthetic µ-map generated by the µ-map generator, (**C**) voxel-wise difference map (B-A), and (**D**) overlay of the segmentation results.

Taken together, the DL algorithm for the automatic kidney segmentation was finalized to be (1) synthetic µ-map input (no SPECT support), (2) widowing-maximum normalization of the µ-map (upper limit of 0.5), (3) GDSC loss function, (4) 3 classes of segmentation, and (5) Residual U-Net architecture with attention module applied (Fig. 6).

### The verification of the CT-free SPECT for GFR

Using the additional 50 kidney SPECT/CT cases as reference (Table 1), the performances of synthetic µ-map generator and automatic kidney segmentation algorithm of the CT-free kidney SPECT was investigated (Table 4). The GFR (mL/min) was employed as the ultimate parameter for the competency of the CT-free kidney SPECT algorithms. High correlation (average R^2^0.9772) and low differences (average MSE, 9.83662E-05; average %NMAE, 1.8446%) were observed for the attenuation coefficients between synthetic µ-maps and ground truth CT-derived µ-maps. For the kidney segmentation results, the automatic DL algorithm showed high agreements (total DSC, 0.818) and low difference (total VD, 17.9 mL), compared with CT-based manual segmentation (Table 4).

**Table 4:** The verification of the CT-free kidney SPECT (*n* = 50 extra cases). (input = µ-map only, normalization = windowing then maximum normalization, loss function = gdsc loss, number of classes = 3, model architecture = residual U-Net with attention module)

Synthetic µ-map generator			Automatic kidney segmentation algorithm					
R^2^	MSE	%NMAE	DSC (total)	Left DSC	Right DSC	VD in mL (total)	VD in mL (left)	VD in mL (right)
0.9772 ± 0.0090 (0.9522–0.9898)	9.8E−05 ± 3.8E-−5 (0.0000405–0.00020191)	1.8446 ± 0.3549 (1.0656–2.7411)	0.818 ± 0.056 (0.513–0.887)	0.788 ± 0.179 (0.000–0.894)	0.812 ± 0.050 (0.602–0.881)	17.9 ± 43.6 (− 62.3–130.5)	10.1 ± 22.4 (− 434.3–70.6)	7.8 ± 26.70 (− 36.5–61.6)

**Fig. 7:**
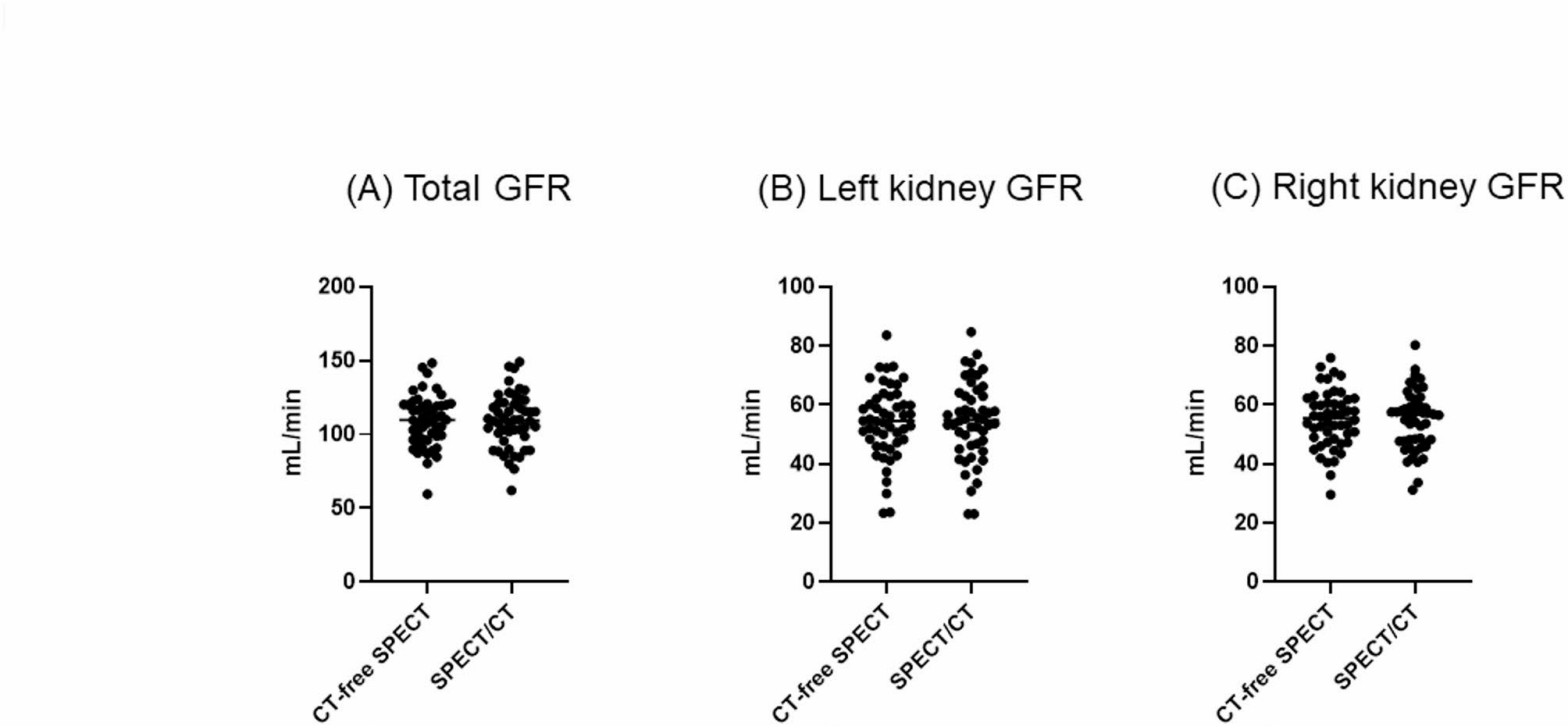
No difference of GFR between CT-free SPECT and conventional SPECT/CT. (**A**) Total GFR, (**B**) Left kidney GFR, and (**C**) Right kidney GFR for CT-free SPECT vs. SPECT/CT, respectively.

In terms of GFR, the CT-free kidney SPECT was not significantly different from the conventional SPECT/CT (Fig. 7). There was no significant difference in individual kidney GFR between CT-free SPECT and conventional SPECT/CT for the right kidney (55.1 ± 9.7 mL/min vs. 54.8 ± 10.0 mL/min, *p* = 0.5041) and the left kidney (54.2 ± 12.4 mL/min vs. 54.4 ± 13.4 mL/min, *p* = 0.5422). Consequently, the total GFR also showed no significant difference between CT-free SPECT and conventional SPECT/CT (109.3 ± 17.3 mL/min vs. 109.2 ± 18.4 mL/min, *p* = 0.9396).

### Advantages of AI-driven CT free SPECT over conventional SPECT/CT

Regarding radiation exposure to patients, conventional SPECT/CT posed a range of effective doses of 3.313–8.563 mSv; the sum of SPECT component (1.813 mSv for Tc-99 m DTPA 370 MBq injection^41^) and CT component [1.5–6.75 mSv using variable dose-length product (100–450 mGy-cm) and a conversion factor of 15 µSv/mGy-cm over the abdomen and pelvis^42^. As CT-free SPECT application eliminates the CT component of radiation exposure, a 45.3–78.8% reduction of radiation exposure could be achieved^32^.

With regard to human resources savings, the AI algorithms for the CT-free SPECT conducted the automatic kidney segmentation in less than a min, whereas manual kidney segmentation took at least 40 min.

## Discussion

Technetium-99 m diethylenetriaminepentaacetic acid (Tc-99 m DTPA) renal scintigraphy has been used to diagnose functional renal disorders. This is because the renal uptake mechanism of Tc-99 m DTPA is dependent on the GFR, a crucial biological measure of renal function. A correlation between the GFR and %ID of Tc-99 m DTPA has been established in multiple studies^7–9^. Although %ID measurement traditionally relied on two-dimensional planar scintigraphy, three-dimensional quantitative SPECT/CT imaging offers greater accuracy and consistency for renal %ID measurements and thus for GFR assessment^10^. Since 2017, our institution has been using Tc-99 m DTPA SPECT/CT for GFR assessment, which was one of the typical quantitative SPECT/CT applications. Two years later, we developed CT-based automatic kidney segmentation technique using CNNs, tremendously reducing human efforts for kidney segmentation^28^. Our most recent development allows CNNs to generate a synthetic µ-map from SPECT data alone, eliminating the need for CT input^32^a method that has been fully applied to CT-free thyroid SPECT imaging^31^. In this study, our primary objective was to establish a CT-free quantification methodology in kidney SPECT. By using only SPECT data, we trained the CNNs to create µ-maps^32^. The automatic kidney segmentation algorithm, currently developed under base of the synthetic µ-map, completed the CT-free GFR SPECT.

With regard to kidney segmentation performance, the DSC of 0.818 of our study may appear slightly inferior to the higher DSCs (0.86–0.91) reported by Jackson et al.^43^. However, the training conditions differed significantly. Our study was conducted in patients with primary renal disease like tumors, stones, or a post-resected state, which presented challenges for kidney contouring. In contrast, Jackson’s study was conducted in patients with normal kidney morphology. The use of synthetic µ-map rather than high resolution CT images may also have contributed to the lower DSC in our study. Nonetheless, the clinical relevance of segmentation using synthetic µ-maps was not compromised, as quantification of renal radioactivity and GFR values remained comparable to those obtained with CT-based segmentation in our validation study.

The CT roles in the conventional quantitative SPECT/CT could be substituted by the DL approach. The use of CT for the target volume estimation is a reasonable approach in the hybrid imaging modality of SPECT/CT. CT-based OS has evolved from manual, to semi-automatic, and to fully automatic methods^28,44^. Moreover, CT has become the preferred method for AC because of its superior image quality, reduced imaging time, and precise tissue delineation compared to radionuclide transmission scans^11,12^. However, a CT-free approach is generally welcomed in terms of reducing patients’ radiation exposure. In the current research, we showed that CT-free kidney segmentation was possible using the synthetic µ-maps generated from the SPECT images. Furthermore, the kidney segmentation process was fully automated, effectively reducing human efforts without compromising the accuracy for GFR measurement (Fig. 7).

We believe that CT-free SPECT, replacing the conventional SPECT/CT, may become a trend in the nuclear medicine practice.

### Limitations

Despite the promising results, this study has some unavoidable limitations. First, although the dataset included scans from two different versions of SPECT/CT systems manufactured by the same vendor, all data were acquired at a single institution. Therefore, the generalizability of our method to other institutions, vendors, or acquisition protocols has not been fully validated. A multi-center study would be necessary to confirm the robustness of this approach in more diverse clinical environments. Second, external validation using public SPECT database was practically impossible. We did not use attenuation/scatter-corrected SPECT images but instead relied on non-corrected SPECT images, which can only be derived from sinograms. Unfortunately, such sinograms are not available in any public database. Third, the current study did not enhance the clinical utility of conventional SPECT imaging. The CT-free SPECT generated by deep-learning did not yield more accurate GFR values than conventional SPECT/CT. It only demonstrated non-inferiority. Therefore, the practical impact on clinical decision-making remains unclear.

## Conclusion

We developed quantitative kidney SPECT technique which did not require CT imaging for the attenuation correction and kidney segmentation. CT-free GFR SPECT, replacing the conventional GFR SPECT/CT, could reduce redundant radiation exposure to patients and save human resources used for just target delineation. AI-assisted CT-free SPECT technique may have an impact on conventional SPECT/CT imaging.

All authors approved the submitted version, and agreed both to be personally accountable for the authors’ own contributions and to ensure that questions related to the accuracy or integrity of any part of the work, even ones in which the authors were not personally involved, are appropriately investigated, resolved, and the resolution documented in the literature.

## Electronic supplementary material

Below is the link to the electronic supplementary material.

Supplementary Material 1

## Data Availability

The datasets generated and/or analyzed during the current study are not publicly available due to restrictions imposed by IRB (Institutional Review Board) but are available from the corresponding author on reasonable request.
