# Supplementary material for: CT-free kidney single-photon emission computed tomography for glomerular filtration rate
Source: Sci Rep. 2025 Jul 25;15:27105. doi: 10.1038/s41598-025-12595-2 (PMC12297543; doi:10.1038/s41598-025-12595-2)
Supplement: Supplementary file 1 — Supplementary Material 1 [file 41598_2025_12595_MOESM1_ESM.docx]

# **SUPPLEMENTARY MATERIAL**

**Acquisition and reconstruction of kidney SPECT/CT**

No dietary restrictions were imposed during the acquisition of the Tc-99m DTPA kidney SPECT/CT imaging. Half an hour before the SPECT/CT imaging, 500 mL of water was provided to the patients for hydration. Patients were positioned on the table of one of two dual-head SPECT/CT scanners (NMCT670 or NMCT670pro; GE Healthcare, Chicago, IL, USA) equipped with low-energy high-resolution collimators. Tc-99m DTPA (TechneScan^R^ DTPA; Mallinckrodt Pharmaceuticals, Dublin, Ireland) was intravenously injected via the antecubital vein at an activity of 370 MBq.

SPECT images were acquired 2–3 min postinjection of Tc-99m DTPA under the following conditions: primary emission energy at 140 keV (20% window: 126–154 KeV), scatter energy at 120 keV (10% window: 115–125 KeV), 1-min continuous acquisition mode with counter-clockwise rotation, no body contour option, and acquisition zoom factor of 1.28. Immediately after the SPECT scan, a helical CT scan was performed using the following parameters: tube voltage of 120 KVp, tube current of 60–210 mA with autoMa function at a noise level of 20, detector collimation of 20 mm (= 16$\times$1.25 mm), helical thickness of 2.5 mm, table speed of 37 mm/s, table feed per rotation of 18.75 mm/rot, tube rotation time of 0.5 s, and pitch of 0.938:1.

SPECT reconstruction was performed using iterative ordered-subset expectation–maximization (OSEM) (4 iterations and 10 subsets; Q.Metrix or Q.VolumetrixMI, GE Healthcare, Chicago, IL, USA). Attenuation correction (AC), scatter correction (SC), and resolution recovery (RR) were applied to the reconstruction of the quantitative SPECT (ACSCRR SPECT). SPECT image matrix was 128x128x128 and voxel size was 3.45$\times$3.45$\times$3.45 mm^3^. CT images were reconstructed into a 512 × 512 × 161 matrix and 0.977 × 0.977 × 2.5 mm^3^ voxel size.

**The phantom study for the determination of the calibration factors**

System sensitivity (calibration factor, CF) was measured using a uniform cylindrical phantom. First, the empty phantom was weighed. Then, it was partially filled with tap water, and a known amount of Tc-99m activity (approximately 1 mCi) was added. The phantom was then completely filled with tap water and weighed again to calculate the total volume based on the weight difference. SPECT imaging was performed using the same acquisition protocol as used in patient scans. This entire procedure was independently repeated three times, and the average value of the calculated system sensitivity was used for analysis. The calibration factors were 151.8 counts/min/μCi for the NMCT670 and 149.3 counts/min/μCi for the NMCT670pro.

**Supplemental Table 1.** Characteristics of the kidney SPECT/CT dataset used for the development of an automatic kidney segmentation algorithm (n=1000)

|  | | For training (n=800) | For validation (n=100) | For testing (n=100) | P value |
| --- | --- | --- | --- | --- | --- |
| Sex (male:female) | | 554:246 | 72:28 | 64:36 | 0.4464 |
| Age (years) | | 56.8±13.4 | 55.9±13.5 | 57.6±11.6 | 0.741077 |
| Height (cm) | | 166.0±9.7 | 167.2±8.4 | 165.3±9.4 | 0.364005 |
| Weight (kg) | | 70.8±13.9 | 70.5±12.0 | 70.1±14.0 | 0.736455 |
| BSA* (m^2^) | | 1.78±0.20 | 1.79±0.18 | 1.77±0.21 | 0.539665 |
| Reason for SPECT/CT | Normal (kidney donor) | 6 | 1 | 3 | 0.4910 |
|  | Renal tumor | 247 (Rt:Lt:Both=118:125:4) | 33 (Rt:Lt:Both=16:17:0) | 27 (Rt:Lt:Both=17:9:1) |  |
|  | Urinary stone | 119 (Rt:Lt:Both=35:44:40) | 15 (Rt:Lt:Both=6:6:3) | 17 (Rt:Lt:Both=6:5:6) |  |
|  | Post partial nephrectomy | 412 (Rt:Lt:Both=216:186:10) | 51 (Rt:Lt:Both=24:26:1) | 53 (Rt:Lt:Both=21:32:0) |  |
|  | Post total nephrectomy | 8 (Rt:Lt=6:2) | 0 | 0 |  |
|  | Others | 8 † | 0 | 0 |  |

* BSA; body surface area by the Dubois formula:

BSA (m^2^) = 0.007184 x weight (kg)^0.425^ x height (cm)^0.725^

† Others include 3 ureter tumors (Rt:Lt=1:2), 3 ureter stricture (Rt:Lt=3:0), and 2 horse-shoe kidneys with renal stone (Rt:Lt=0:2).

Data are mean±standard deviation.


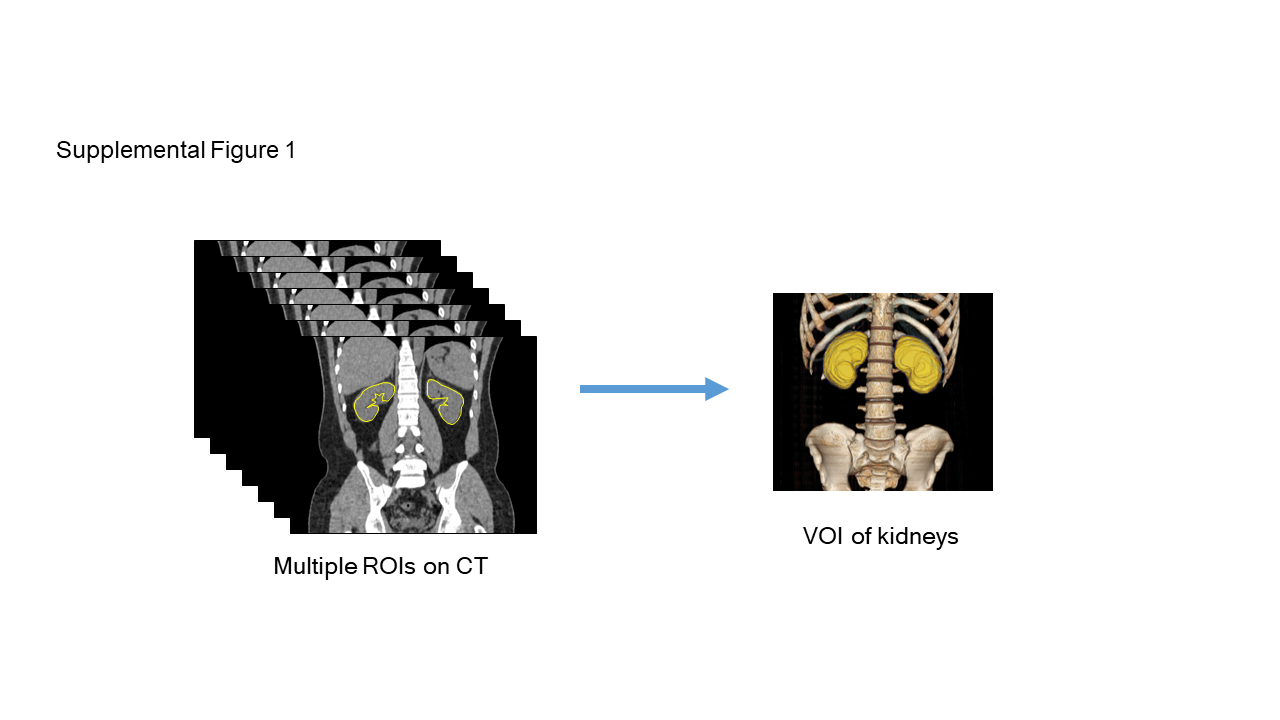


Supplemental Figure 1. How to segment kidneys. The manual process of drawing of regions-of-interest (ROIs) to generate volumes-of-interest (VOIs) for both kidneys takes approximately 40 minutes.


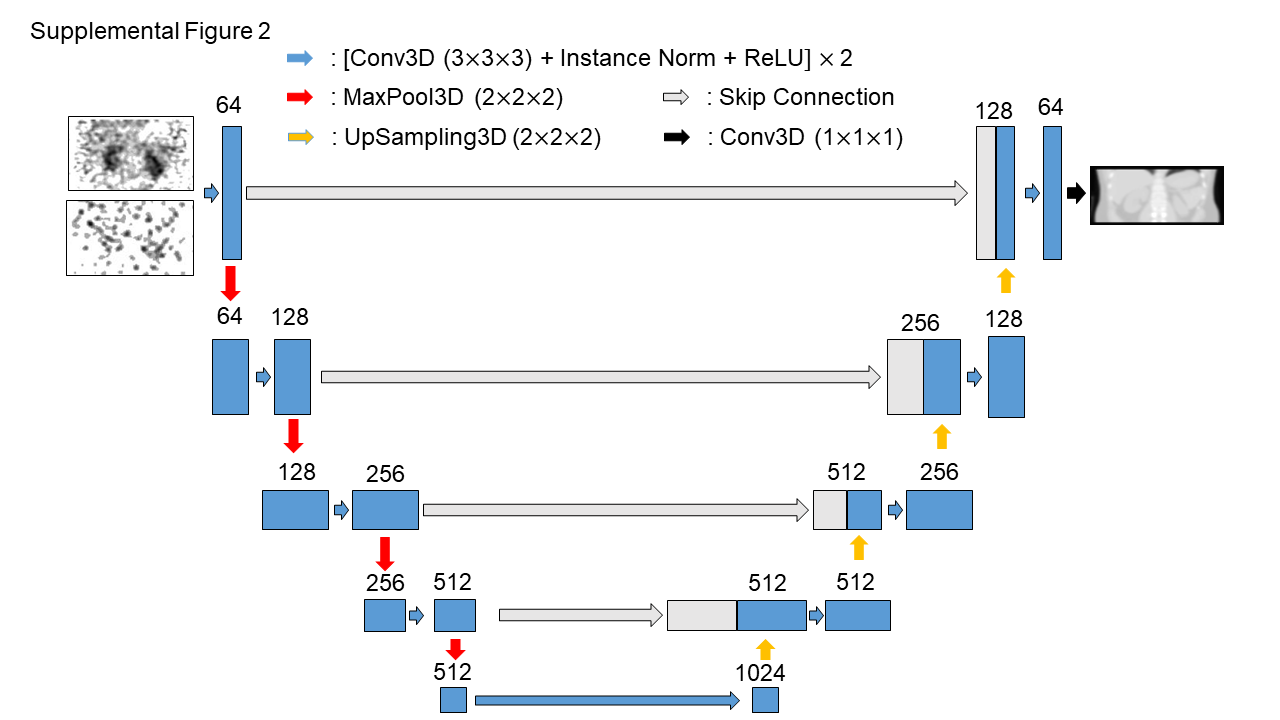


Supplemental Figure 2. Model architecture for the μ-map generation.


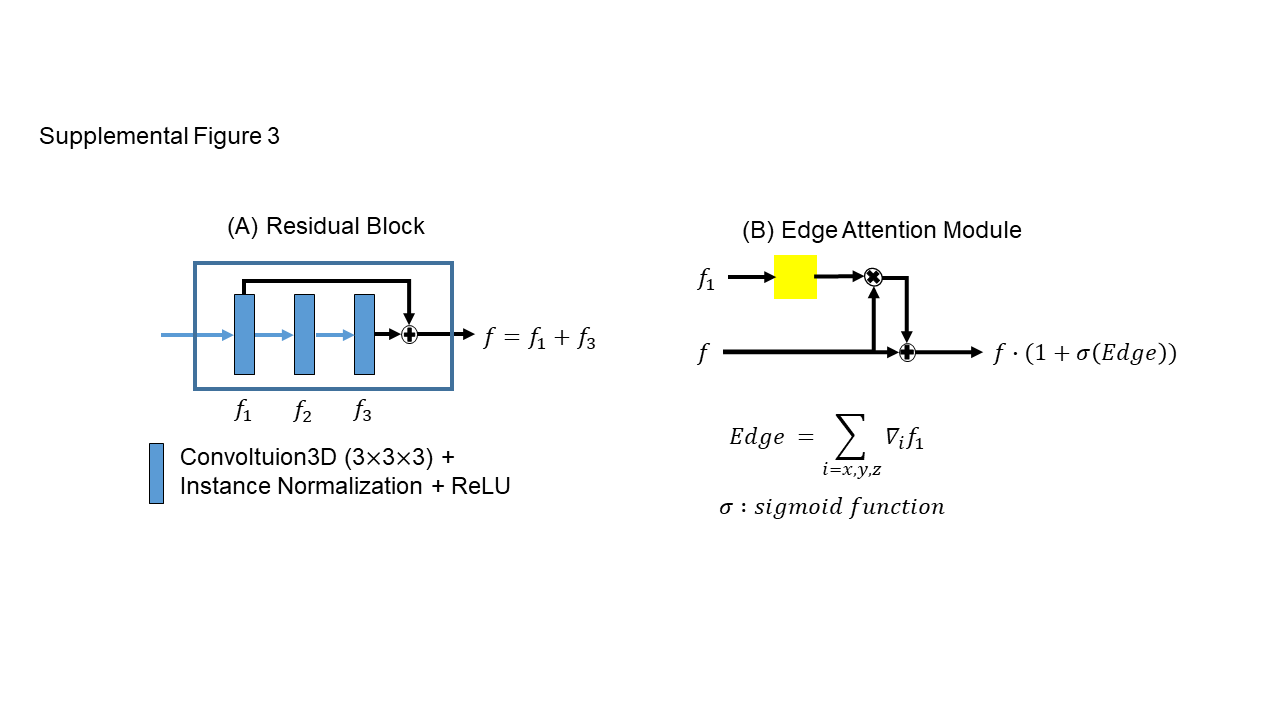


Supplemental Figure 3. (A) Residual block and (B) edge attention module.

**Evaluation of outcomes**

Dice similarity coefficient (DSC) was defined as follows:

$$DSC((G\left( X \right),Y)=\frac{2\times\left| G\left( X \right)\cap Y \right|}{\left| G(X) \right|+\left| Y \right|}$$

where Y is the manual kidney segmentation map as ground truth, G(X) is the AI-driven segmentation map from the synthetic μ-map input X, and G(X)∩Y is the element-wise product of G(X) and Y.

*R^2^*, mean square error (MSE), and %normalized mean absolute error (%NMAE) were defined as follows:

$$R^{2}=1-\frac{\sum{((G\left( X \right)-Y)}^{2}}{\sum{(Y-\bar{Y})}^{2}}$$

$$MSE=\frac{1}{No. of Voxels}\sum{((G\left( X \right)-Y)}^{2}$$

$$\%NMAE=\frac{1}{No. of Voxels}\sum\frac{\left| G\left( X \right)-Y \right|}{\max\left( Y \right)-min(Y)}$$

where Y is the ground truth (i.e., original μ-map), $\bar{Y}$ is the mean of Y, and G(X) is the synthetic μ-map from the SPECT input X.

**Normalization of the SPECT support**

The equation for the maximum normalization is:

$$V_{norm}=\frac{V_{origin}-V_{min}}{V_{max}-V_{min}}$$

The equation for the logarithmic maximum normalization is:

$$V_{norm}=\frac{{log}_{10}(1+V_{origin}-V_{min})}{{log}_{10}(1+V_{max}-V_{min})}$$

where V_norm_, V_origin_, V_max_ and V_min_ represent the voxel values for the normalized, original, maximum, and minimum values, respectively.

**Supplemental Table 2.** Performance by SPECT support (n=100 testing cases). (CNN model = U-Net, loss function = CCE loss, number of classes = 3)

| Input | Normalization for SPECT | DSC (total) | DSC (left) | DSC (right) | VD in mL (total) | VD in mL (left) | VD in mL (right) |
| --- | --- | --- | --- | --- | --- | --- | --- |
| ATT+P | Max | 0.798±0.091 (0.122-0.880) | 0.789±0.127 (0.000-0.892) | 0.793±0.128 (0.000-0.907) | 18.0±44.0 (-76.9-200.7) | 15.6±23.4 (-39.4-104.1) | 2.4±25.7 (-77.1-96.6) |
| ATT+PS | Max | 0.802±0.063 (0.510-0.881) | 0.791±0.107 (0.000-0.892) | 0.797±0.119 (0.000-0.899) | 18.8±44.2 (-95.2-140.1) | 12.9±23.2 (-56.3-87.0) | 5.9±25.4 (-57.4-110.9) |
| ATT+Q | Max | 0.809±0.059 (0.503-0.886) | 0.799±0.097 (0.066-0.903) | 0.805±0.106 (0.158-0.919) | 4.1±43.6 (-114.9-138.1) | 10.0±21.2 (-39.4-83.3) | -5.9±27.4 (-77.1-116.2) |
| ATT+P | Log-max | 0.772±0.101 (0.197-0.885) | 0.752±0.159 (0.000-0.913) | 0.773±0.138 (0.000-0.911) | 32.7±73.1 (-113.9-276.0) | 22.3±41.2 (-316.7-189.4) | 10.4±35.6 (-129.6-128.6) |
| ATT+PS | Log-max | 0.769±0.112 (0.015-0.875) | 0.765±0.134 (0.004-0.894) | 0.751±0.184 (0.000-0.914) | 34.9±60.2 (-79.6-218.3) | 17.7±31.5 (-38.3-134.2) | 17.1±33.3 (-47.3-128.6) |
| ATT+Q | Log-max | 0.699±0.099 (0.425-0.853) | 0.648±0.128 (0.270-0.852) | 0.757±0.091 (0.431-0.891) | -106.9±109.0 (-446.3-114.7) | -82.1±77.2 (-316.7-76.1) | -24.8±37.8 (-129.6-68.7) |

ATT: Attenuation map (μ-map)

P: Primary emission SPECT

S: Scattering SPECT

Q: Quantitative SPECT

Max: maximum normalization

Log-Max: logarithmic maximum normalization

DSC: Dice similarity coefficient

VD: volume difference

Date are mean±standard deviation (range).

**Windowing-maximum normalization of the μ-map**

We attempted to find the optimal upper limit of the μ-map attenuation coefficients for the windowing-maximum (wind-max) normalization. With the lower limit set to 0, the upper limits of μ-map attenuation coefficients (i.e., wind-max factor) were restricted to 0.3, 0.4, or 0.5, and then maximum normalization was applied. As a result, the wind-max normalization had the most optimal performance with the 0.5 wind-max factor, showing the highest DSC and the lowest VD.

**Supplemental Table 3.** Performance by windowing-maximum normalization of the μ-map input (n=100 testing cases) (input = μ-map only, CNN model = U-Net, loss function = CCE loss, number of classes = 3)

| Wind-max factor | DSC (total) | Left DSC | Right DSC | VD in mL (total) | VD in mL (left) | VD in mL (right) |
| --- | --- | --- | --- | --- | --- | --- |
| 0.5 | 0.801±0.059 (0.511-0.886) | 0.796±0.076 (0.375-0.897) | 0.801±0.076 (0.444-0.900) | 7.4±40.1 (-4.6-0.0) | 6.7±20.1 (-2.1-2.9) | 0.7±23.4 (-2.5-0.0) |
| 0.4 | 0.796±0.066 (0.490-0.883) | 0.791±0.106 (0.000-0.891) | 0.784±0.127 (0.009-0.894) | 19.9±43.4 (-3.8-0.0) | 10.9±22.9 (-1.6-3.6) | 8.9±26.5 (-2.4-0.0) |
| 0.3 | 0.799±0.062 (0.489-0.881) | 0.791±0.102 (0.029-0.887) | 0.792±0.105 (0.100-0.886) | 14.4±41.7 (-3.5-0.0) | 8.4±22.1 (-1.5-3.5) | 6.0±24.5 (-2.1-0.0) |

Wind-max: windowing then maximum normalization

CCE: categorical cross-entropy

DSC: Dice similarity coefficient

VD: volume difference

Date are mean±standard deviation (range).
